# Supplementary material for: Tuberculosis Severity Predictive Model Using Mtb Variants and Serum Biomarkers in a Colombian Cohort of APTB Patients
Source: Biomedicines. 2023 Nov 22;11(12):3110. doi: 10.3390/biomedicines11123110 (PMC10740695; doi:10.3390/biomedicines11123110)
Supplement: Supplementary file 1 [file biomedicines-11-03110-s001.zip › biomedicines-2633424-supplementary.pdf]

Figure S1

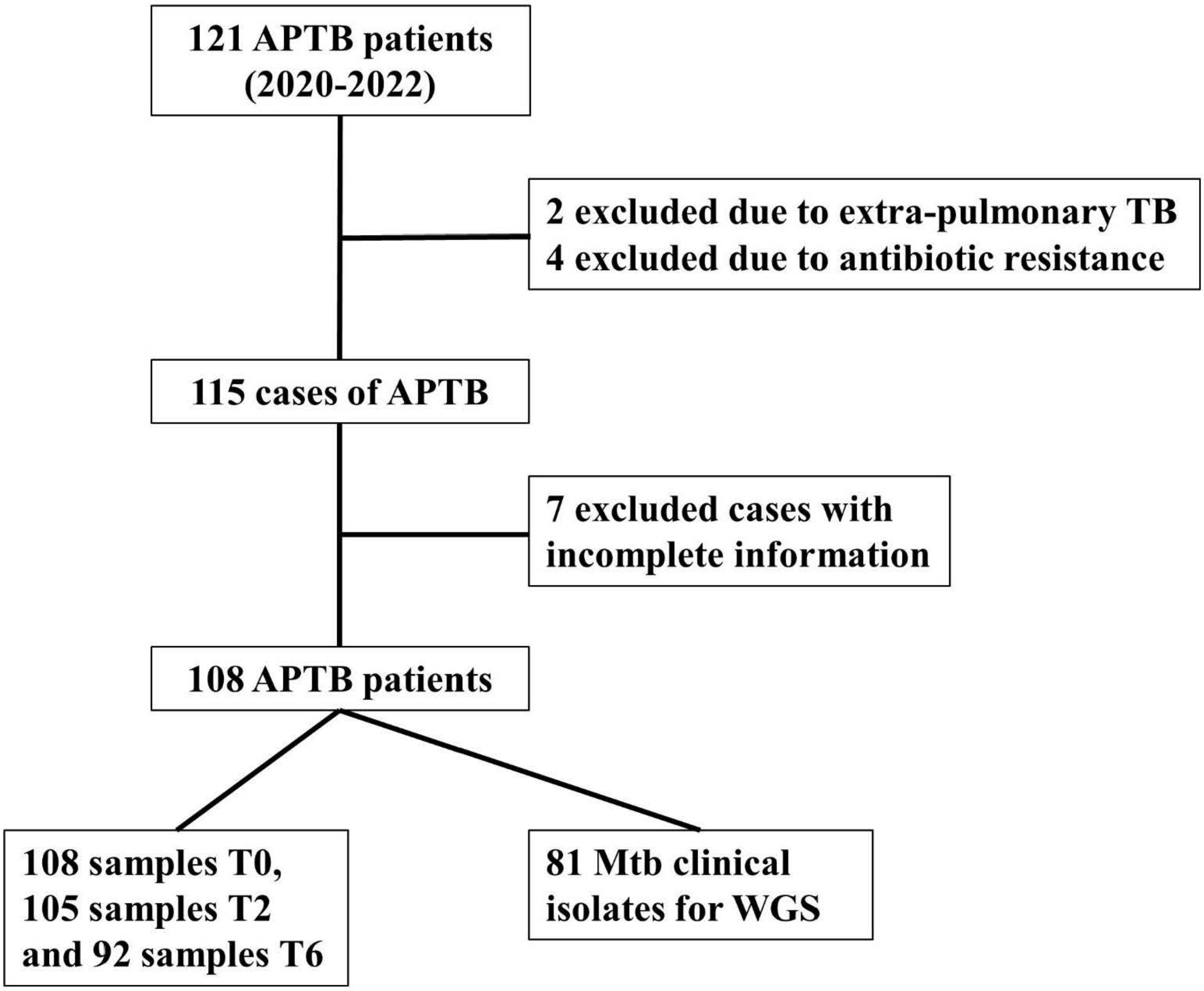
121 APTB patients
(2020-2022)
2 excluded due to extra-pulmonary TB
4 excluded due to antibiotic resistance
115 cases of APTB
7 excluded cases with
incomplete information
108 APTB patients
108 samples T0,
105 samples T2
and 92 samples T6
81 Mtb clinical
isolates for WGS

Table S1. TB score GICG classification

Parameters	Points assigned
Self-reported	
Cough	1
Hemoptysis	3
Dyspnea	1
Chest pain	1
Night sweating	1
Anemic conjunctivae	1
Tachycardia	1
Positive finding at lung auscultation	1
Axillary temperature >37.0°C	1
BMI*<18	2
BMI<16	2
MUAC**<220	2
MUAC<200	2
*BMI: Body Mass Index; **MUAC: Middle Upper Arm Circumference.	
**TB score I**	0-4
**TB score II**	5-8
**TB score III**	≥ 9

Table S2. ROC curve values

Cytokines	APTB mild vs severe			
	Cut point	*AUC (IC 95%)	Sensibility	Specificity
**IL-6**	3.92 (pg/mL)	0.82 (0.62-1)	0.86	0.66
**IFN-γ**	7.36 (pg/mL)	0.79 (0.59-0.99)	0.73	0.80
**IL-33**	83.79 (pg/mL)	0.81 (0.62-0.99)	0.78	0.83
**CHIT-1**	13.25 (ng/mL)	0.75 (0.61-0.89)	0.82	0.69

*AUC: Area under the curve.
The ROC curve values were calculated using the Youden index.

Table S3 . Gene ontology (GO) categories.

Molecular function	GO term	Description	Genes included	Strength	False Discovery Rate (FDR)
**Biological process**	GO:0008556	ATPase K+ transporter activity	3 of 3	2.91	1.45e-05
**Biological process**	GO:0010447	Response to acid pH	6 of 16	2.4	2.13e-11
**Biological process**	GO:0019432	Triglyceride biosynthetic process	2 of 17	1.9	0.0482
**Biological process**	GO:0071731	Nitric oxide response	2 of 19	1.85	0.0482
**Biological process**	GO:0006071	Glycerol metabolic process	2 of 22	1.79	0.0482
**Cellular component**	GO:0005887	Integral component of the plasma membrane	3 of 110	1.34	0.0371
**Cellular component**	GO:0016021	Integral component of the plasma membrane	4 of 388	0.92	0.0371
**Key words Uniprot**	**Description**		**Genes included**	**Strength**	**False Discovery Rate (FDR)**
**KW-0633**	Potassium transport		3 of 5	2.68	1.30e-05
**KW-0902**	Two component system		2 of 29	1.74	0.0286
**KW-0597**	Phosphoprotein		3 of 87	1.44	0.0066
**KW-1003**	Cellular membrane		4 of 462	0.84	0.0338
**Vías KEGG**	**Description**		**Genes included**	**Strength**	**False Discovery Rate (FDR)**
**mtv02020**	Two component system		5 of 162	1.4	1.58e-05
**Protein domain and characteristics (Inter-PRO)**	**Description**		**Genes included**	**Strength**	**False Discovery Rate (FDR)**
**IPR000084**	PE-PGRS family		12 of 88	0.69	0.0192
